# Investigation of Cytokine Changes in Osteoarthritic Knee Joint Tissues in Response to Hyperacute Serum Treatment

**DOI:** 10.3390/cells8080824

**Published:** 2019-08-03

**Authors:** Dorottya Kardos, Bence Marschall, Melinda Simon, István Hornyák, Adél Hinsenkamp, Olga Kuten, Zsuzsanna Gyevnár, Gábor Erdélyi, Tamás Bárdos, Tamás Mirkó Paukovits, Krisztián Magos, György Béres, Kálmán Szenthe, Ferenc Bánáti, Susan Szathmary, Stefan Nehrer, Zsombor Lacza

**Affiliations:** 1 Institute Clinical Experimental Research, Semmelweis University, Budapest 1094, Hungary; 2 Center for Regenerative Medicine, Danube University, Krems-an-der-Donau 3500, Austria; 3 Institute Sport and Health Sciences, University of Physical Education, Budapest 1123, Hungary; 4 Kastelypark Clinic, Tata 2980, Hungary; 5 RT-Europe Nonprofit Research Center, Mosonmagyaróvár 9200, Hungary; 6 Galenbio Ltd., Mosonmagyaróvár 9200, Hungary; 7 Orthosera GmbH, Krems an der Donau 3500, Austria

**Keywords:** hyperacute serum, knee osteoarthritis, inflammatory cytokines, matrix metalloproteases, bone remodeling

## Abstract

One option to fight joint degradation and inflammation in osteoarthritis is the injection of activated blood products into the synovial space. It has been demonstrated that hyperacute serum is the most proliferative among plasma products, so we investigated how the cytokine milieu of osteoarthritic knee joint reacts to hyperacute serum treatment in vitro. Cartilage, subchondral bone, and synovial membrane explanted from osteoarthritic knees were stimulated by interleukin-1 beta (IL-1β) and the concentration of 39 biomarkers was measured in the co-culture supernatant after hyperacute serum treatment. The IL-1β stimulation triggered a strong inflammatory response and enhanced the concentrations of matrix metalloproteinase 3 and 13 (MMP-3 and MMP-13), while hyperacute serum treatment reduced inflammation by decreasing the concentrations of IL-1β, tumor necrosis factor alpha (TNF-α), interleukin-6 receptor alpha (IL-6Rα), and by increasing the level of interleukin-1 antagonist (IL-1RA) Cell viability increased by day 5 in the presence of hyperacute serum. The level of MMPs-1, 2, and 9 were higher on day 3, but did not increase further until day 5. The concentrations of collagen 1 alpha 1 (COL1A1) and osteonectin were increased and receptor activator of nuclear factor kappa-B ligand (RANKL) was reduced in response to hyperacute serum. We concluded that hyperacute serum treatment induces cell proliferation of osteoarthritic joint tissues and affects the cytokine milieu towards a less inflamed state.

## 1. Introduction

**Figure 1:**
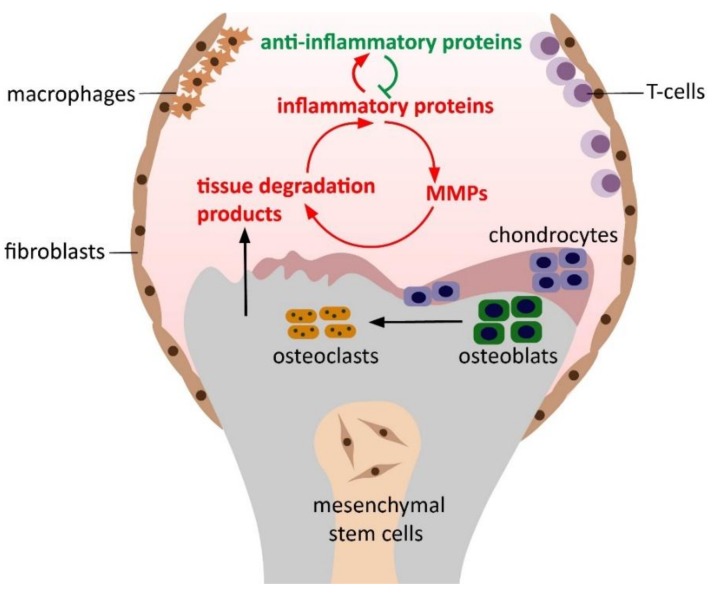
Pathogenesis of osteoarthritis, including the progression of the osteoarthritis (OA) positive feedback loop, including synovial inflammation and tissue degradation. Inflammatory proteins produced mainly by chondrocytes, synovial fibroblasts, and macrophages induce the excess production of tissue-degrading enzymes, such as matrix metalloproteinases. Products of cartilage breakdown are phagocytosed by the synovial cells triggering the release of even more proinflammatory proteins.

Osteoarthritis (OA) is the most common degenerative joint disorder characterized by cartilage degradation, subchondral bone sclerosis, the formation of osteophytes, and synovial inflammation [1]. Bone, cartilage, and synovium communicate via soluble mediators secreted into the synovial fluid, thus, the disease affects the whole joint, despite the traditional view that OA is a cartilage-only disorder [2]. It is unknown yet, whether synovial inflammation is the primary effect of the disease or whether it is the result of the cartilage degradation and subchondral bone lesion [3]. Nonetheless, inflammatory mediators, proteolytic enzymes, cartilage, and bone degradation proteins detected in the synovial fluid can be used as potential biomarkers of OA [4]. The two main inflammatory cytokines of OA are interleukin-1 beta (IL-1β) and tumor necrosis factor alpha (TNF-α), produced mainly by chondrocytes, synovial fibroblasts, and macrophages. IL-1β and TNF-α induce chondrocyte, synovial, and immune cells to produce a broad spectrum of inflammatory proteins, such as IL-2, IL-6, IL-12, IL-15, IL-17, IL-18, and IL-21. In addition, they evoke apoptosis of chondrocytes and synovial fibroblasts and inhibit synthesis of collagen-type II and aggrecan [5]. Most of these cytokines trigger the production of chemokines and other proinflammatory proteins, such as resistin and oncostatin M (OSM), further enhancing synovitis, inducing excess production of several matrix metalloproteinases (MMPs) and other proteolytic enzymes, decreasing the expression of inhibitory proteins of matrix metalloproteinases (TIMPs) [6,7,8]. Matrix metalloproteinases, most predominantly MMP-1, -2, -3, -9, and -13, begin degradation of the extracellular matrix of cartilage and bone; moreover, they contribute to the enhanced production of receptor activator of nuclear factor kappa-B ligand (RANKL) by osteoblasts and chondrocytes, which results in excessive osteoclast formation and bone resorption [9,10,11,12,13]. Products of cartilage breakdown are phagocytosed by the synovial cells triggering the release of even more proinflammatory proteins [14]. Thus, a positive feedback loop develops involving synovial inflammation and tissue degradation [15]. In response, anti-inflammatory cytokines such as IL-1 receptor antagonist (IL-1RA), IL-4, IL-10 and IL-13 are released to prevent tissue degradation and synovitis (see Figure 1) [16].

One may hypothesize that changing the milieu of the joint by adding a fresh set of anti-inflammatory cytokines may tip the balance towards regeneration [17,18]. Since injury invokes a regenerative cytokine response from blood, it is possible to harness this mechanism to fight joint degradation by injecting activated blood products such as platelet rich plasma (PRP) into the synovial space [19]. Several studies showed, that PRP and other blood derivatives have beneficial effects on pain and mobility when injected into the osteoarthritic knee joint at various stages of degradation [20,21,22]. Activated platelets secrete growth factors, such as transformation growth factor beta (TGF-β), platelet derived growth factor (PDGF), platelet factor-4 (PF-4), and different cytokines, which attract tissue specific progenitor cells and mesenchymal stem cells to promote regeneration. However, translation of these cellular level studies to clinical benefit is not straightforward and several large and well-controlled studies failed to confirm the positive effects of PRP seen in the pilot studies [23,24,25]. One explanation for this discrepancy is that there is a wide variation among activated blood derivatives in composition and biological action [26]. Our previous studies investigated the effects of plasma and serum products on osteoarthritic bone and cartilage cells, as well as mesenchymal stem cells from the subchondral bone. We consistently observed, that the serum fraction of platelet rich fibrin, which is essentially the serum harvested at a hyperacute phase, is the best supporting agent for cell proliferation without affecting lineage [27,28]. As an intermediate solution between cell cultures and clinical investigations, we designed an in vitro joint explant model, that contains all three tissues of the joint as a three-dimensional unit, i.e., cartilage, synovial membrane and subchondral bone explant co-cultures. The aim of the present study was to investigate the effect of IL-1β induction and hyperacute serum treatment on the cytokine profile of a complex osteoarthritic joint model in vitro.

## 2. Materials and Methods

### 2.1. Hyperacute Serum Isolation

Blood samples were obtained from healthy donors of both sexes aged 24–45 years (Institutional Review Board (IRB) approval number 33106-1/2016/EKU). Eighteen milliliters of venous blood was taken by venipuncture from the volunteers with the hypACT Inject device (OrthoSera GmbH, Krems, Austria) [29]. The device was immediately centrifuged at 1710 relative centrifugal force (rcf)× for 8 min. After centrifugation the red blood cell containing fraction was removed and hyperacute serum was pressed out from the clotted platelet rich fibrin under a laminar flow tissue culture hood. The serum was either used fresh or stored at −20 °C until use. Serum samples were pooled from five donors in order to reduce inter-donor variability.

### 2.2. Tissue Culture

**Figure 2:**
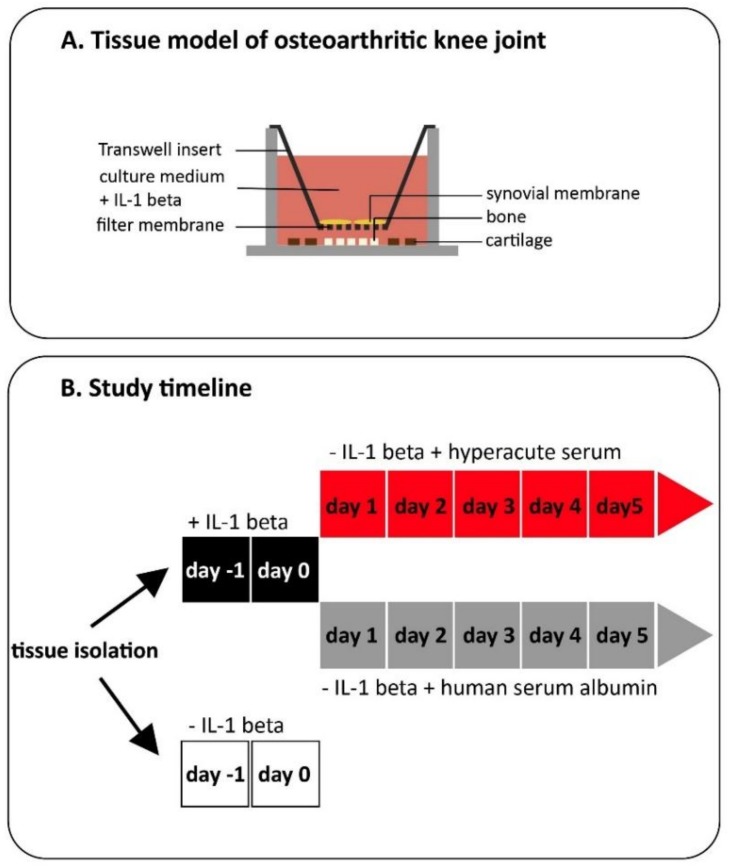
Design of the experiment. Our in vitro knee tissue co-culture model was built from the three main tissue types (i.e., synovial membrane, subchondral bone, and hyaline cartilage), which play roles in the pathogenesis of OA. The structure of the model allows similar communication pathways as it happens in vitro. (**A**) After building the model, tissues were stimulated by IL-1β for 2 days or cultured without IL-1β as a negative control. Medium was exchanged in the IL-1β-stimulated cultures and tissues were treated with 10% hyperacute serum for 5 days. We used human serum albumin with a matched albumin content as a negative control for 5 days (**B**).

Subchondral bone, hyaline cartilage, and synovial membrane were harvested from surgically excised tissues during a routine knee replacement procedure (IRB approval number 33106-1/2016/EKU). We only obtained tissues for research purposes that would have been discarded otherwise. Tissue samples were further processed under sterile conditions in a laminar flow cabinet. Cartilage was harvested and cut from subchondral bone by a sterile biopsy punch (d = 5 mm) into small disks. The marrow of subchondral bone was cut into 3 mm cubes by sterile scalpel, while synovial membrane samples were also cut into small pieces by sterile biopsy punch (d = 5 mm). After harvesting the tissue samples, 5 pieces of bone and 4 pieces of cartilage per well were put into the bottom of a low-attachment 12 well plate (Sigma–Aldrich, St. Louis, MO, USA). The 2 pieces of synovial membrane per well were placed into transwell inserts with 0.4 µm pore polyester membrane (Corning, Corning, NY, USA) to simulate the spatial arrangement of the joint and tissues were co-cultured for 7 days (Figure 2).

Tissues were cultured in 4 mL/well Dulbecco’s modified Eagle’s medium Ham’s F-12 1:1 Mix (DMEM/F-12) containing 12 mM Hepes and L-Glutamine (Lonza, Walkersville, MD, USA), 50 µg/mL l-ascorbic acid-2-phosphate (Sigma–Aldrich, St. Louis, MO, USA), 2 µl/mL Primocin (Invitrogen, Carlsbad, CA, USA) supplemented with 5% human albumin solution (Biotest, Dreieich, Germany) in the presence of 10 ng/mL interleukin 1 beta (IL-1β) (Sigma–Aldrich, St. Louis, MO, USA) in order to keep up the active phase of osteoarthritis in the in vitro setting. Tissue samples without IL-1β stimulation were also co-cultured for 2 days as a negative control.

Experiments were started after a 2 days equilibration period when tissue culture medium was withdrawn and replaced by 10% hyperacute serum containing medium in case of the treated group and with human serum albumin containing medium with a matching albumin content as in the treated group (Figure 2).

Supernatants were collected after IL-1β treatment (day 0), and after hyperacute serum treatment (days 3 and 5) and stored at −80 °C before cytokine measurements. The experiments were performed 12 times with tissues from four different patients (from both genders, aged between 50–70).

### 2.3. Synovial fluid isolation from OA patients

Synovial fluid was harvested from patients who underwent intraarticular knee injection treatments that included the withdrawal of the synovial fluid effusion (IRB approval number 6906/2017/EKU). The samples were immediately frozen and stored at −80 °C until cytokine measurements.

### 2.4. Tissue Viability Measurements

Tissue viability was determined after IL-1β stimulation (day 0) and after hyperacute serum treatment (days 3 and 5) using the Cell Proliferation Kit II (XTT; Roche, Mannheim, Germany) according to the manufacturer’s instructions. Absorbance was measured on days 3 and 5 after 4 hours’ incubation in the staining solution using a PowerWave microplate spectrophotometer (BioTek, Winooski, VT, USA) at 480 nm with a reference wavelength at 650 nm. The optical density results were normalized with the dry weight of each explant.

### 2.5. Cytokine Measurements

A literature review on cytokines in osteoarthritic synovial fluid was conducted and 39 factors (aggrecan, chemokine CC motif ligand 3 (CCL-3), C-X3-C motif chemokine ligand 1 (CX3CL1/fractalkine), interferon gamma (IFN-gamma), IL-12, IL-17a, IL-2, IL-33, MMP-1, resistin, RANKL, CCL-1, cluster of differentiation 163 (CD163), chemokine C-X-C motif ligand 10 (CXCL-10), IL-1ß, IL-13, IL-18, IL-23, IL-6 receptor alpha (IL-6Rα), MMP-2, osteonectin, CCL-2, collagen I alpha 1 (COL1A1), IL-10, IL-15, IL-1 receptor antagonist (IL-1RA, IL-31, leukemia inhibitor factor (LIF), oncostatin M (OSM), TNF-α, MMP-3, CXCL-8/IL-8, IL-4 receptor alpha (IL-4Rα), MMP-13, CCL-5, IL-5, vascular endothelial growth factor A (VEGF-A), IL-22, IL-7, and MMP-9) were identified, which were described to play a role in OA [4,14,16,30,31,32,33]. A custom two-plate Human Magnetic Luminex Assay (R&D System Inc., Minneapolis, Canada) was designed and all 39 factors were quantified in the biological samples by Magpix Luminex Xmap Technology (Thermofisher Scientific, Waltham, MA, USA). Cytokine profiles of the in vitro culture supernatant after IL-1β stimulation and the synovial fluid of OA patients were compared to validate our OA tissue model. Concentrations of the different cytokines in the culture supernatant on days 0, 3, and 5 were compared to each other.

### 2.6. Statistical Analysis

The dataset was normally distributed on the basis of the D’Agostino and Pearson omnibus normality test, thus, the data were analyzed by Pearson correlation test and one-way analysis of variance (ANOVA) and Tukey’s post-hoc test. The correlation was considered to be very strong when *r* > 0.75 and strong when 0.75 > *r* > 0.5. Prism 8 software (Irvine, CA) was used for statistical analysis. Significance level was *p* < 0.05. Data are presented as mean + SEM.

## 3. Results

**Figure 3:**
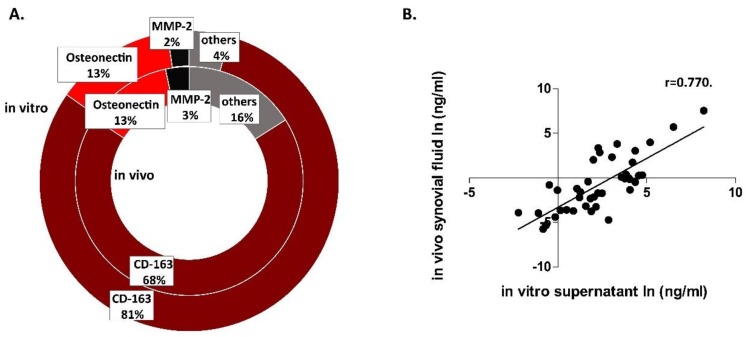
Cytokine pattern comparison. The measured 39 cytokines were present both in vivo and in vitro in a similar distribution. The highest concentrations were measured in the case of cluster of differentiation 163 (CD163), osteonectin, and matrix metalloproteinase-2 (MMP-2) both in vitro and in vivo (**A**). After transforming the dataset, we found a very strong correlation between the cytokine content of the in vitro and in vivo samples: n_synovial fluid_ = 19, n_supernatant_ = 12 (**B**).

The concentration of the 39 different cytokines was measured both in the culture supernatant after IL-1β treatment and in the synovial fluid of OA patients (Figures 5–8 and Figure S1). In order to validate the model, we compared the cytokine composition of the OA synovial fluid with those that were released into the co-culture supernatant by the explants. The level of the cytokines was expressed in % compared to the summarized data of the measured concentrations that was considered to be 100%. The most abundant protein was CD163, followed by osteonectin and MMP-2. (Figure 3). The rest of the molecules was also detectable in both types of the samples, but in a lower concentration. The correlation between the cytokine concentrations of the in vitro and in vivo samples was investigated by Pearson correlation test after transforming the dataset by logarithm because of the outliers (CD-163, MMP-2, osteonectin). A strong correlation (*r* = 0.770) was found between in vitro and in vivo samples, indicating that the in vitro joint model was a fair approximation of the in vivo osteoarthritic joint in terms of cytokine production (Table S1).

**Figure 4:**
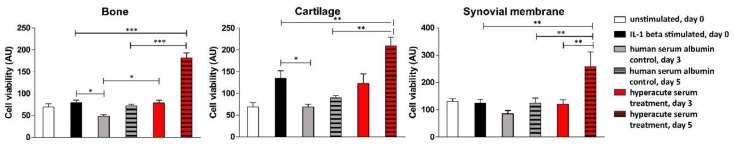
Cell viability in bone, cartilage, and synovial membrane. Hyperacute serum treatment had a positive effect on the cell viability of cartilage, bone, and synovial membrane tissues during a 5 days long culture period, *n* = 12. The significance level was *p* < 0.05, where * means that the *p*-value was between 0.05 and 0.01, ** means that the *p*-value was between 0.01 and 0.001, and *** means that *p*-value was lower than 0.001.

After the withdrawal of IL-1β on day 0, tissues were supplemented with hyperacute serum or human serum albumin containing culture medium and viability was measured further on days 3 and 5. On day 5, the overall viability of the hyperacute serum-treated explants doubled in each tissue type compared to day 0 and to the albumin-treated control, reflecting an ongoing proliferation of cells (Figure 4). The number of cells in the albumin-containing control group did not increase compared to IL-1β treated group and the protein levels were also unchanged. The difference between the tissue viability of albumin-treated and the hyperacute serum-treated samples was significant, thus, a reliable comparison of protein concentrations in the treated and control groups without considering cell proliferation changes is hard to interpret (Table S2).

**Figure 5:**
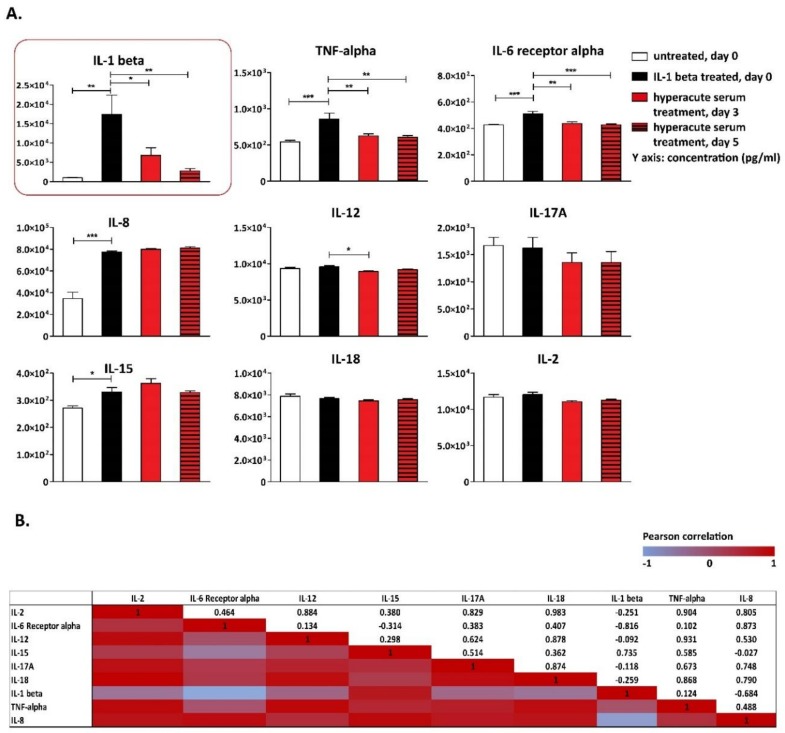
Main osteoarthritic inflammatory cytokines detected in the tissue culture supernatant. IL-1β, IL-6Rα, TNF-α, IL-8, and IL-15 significantly increased in response to IL-1β stimulation. A significant decrease was observed in the concentration of IL-1β and IL-6Rα under the influence of hyperacute serum treatment on day 5. The pattern of TNF-α, IL-2, -12, -17, and -18 was the same; however, changes did not reach the significance level, *n* = 12. The significance level was *p* < 0.05, where * means that the *p*-value was between 0.05 and 0.01, ** means that *p*-value was between 0.01 and 0.001, and *** means that the *p*-value was lower than 0.001 (**A**). The correlations of cytokine changes are presented in heatmap tables. Between day 0 and day 5, the correlations were strong between IL-17, IL-12, and TNF- α, as well as between IL-8, IL-18, and TNF-α, while very strong correlations were found between IL-2 and IL-6Rα, IL-8, IL-12, IL-15, IL-17A, IL-18, TNF-α, *n* = 12 (**B**).

**T**he concentrations of the main inflammatory cytokines and growth factors (i.e., IL-1β, IL-6Rα, TNF-α, and IL-8 and IL-15) significantly increased in response to IL-1β stimulation; however, the extreme increase in IL-1β concentration resulted from both the externally added 10 ng/mL IL-1β and from that produced by the tissues themselves (Figure 5A). Before hyperacute serum treatment, the culture medium was exchanged. During the next 5 days, the measured IL-1β was secreted by the tissues in culture. A significant decrease was observed in the concentrations of IL-1β, TNF-α, and IL-6Rα under the influence of the hyperacute serum treatment (Figure 5A). The trends in IL-2, -12, -17, -18 were similar; however, changes did not reach the level of significance.

**T**he correlation among cytokine changes was investigated from day 0 to day 5 in response to hyperacute serum treatment ((day 0, IL-1+) − (day 5, hyperacute treatment)). Very strong correlations were found between IL-2 and all the main inflammatory cytokines except IL-1β, which was externally added on day 0 to the samples, thus, these results cannot be interpreted. Correlations were also found to be very strong between IL-6Rα and Il-8, IL-12 and IL-18, IL-12 and TNF-α, IL-8A and TNF-α, IL-8 and IL-18, while strong correlations were found between IL-12 and IL-17A, IL-15 and TNF-α, IL-17A and TNF-α, IL-12 and IL-8 (Figure 5B).

**Figure 6:**
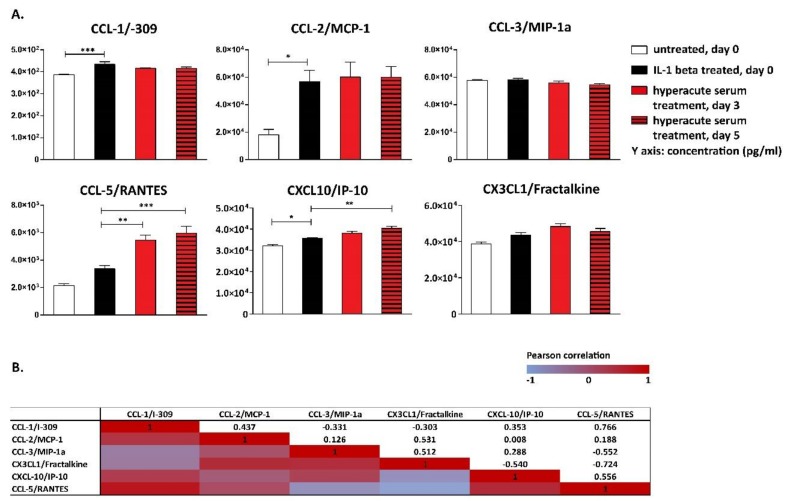
Inflammatory chemokines detected in the tissue culture supernatant. Chemokine CC ligand 1 (CCL-1), CCL-2, and chemokine C-X-C ligand 1 (CXCL-10) increased significantly after IL-1β stimulation, and the level of CCL-5 and CXCL-10 increased even further in response to hyperacute serum, *n* = 12. The significance level was *p* < 0.05, where * means that the *p*-value was between 0.05 and 0.01, ** means that the *p*-value was between 0.01 and 0.001, and *** means that the *p*-value was lower than 0.001 (**A**). The correlations of chemokine changes are presented in heatmap tables. Between day 0 and day 5, strong correlations were present between CCL-5 and CCL-1, factalkine and CCL-2, CCL-3, CCL-5, and CXCL-10, while strong reverse correlations were found between CXCL-10 and fractalkine, CCL-5 and CCL-3, and fractalkine and CCL-5, *n* = 12 (**B**).

Among inflammatory chemokines, CCL-1, CCL-2, and CXCL-10 increased significantly after IL-1β stimulation (Figure 6A). The level of CCL-5 and CXCL-10 increased even further in response to hyperacute serum, while the concentration of CCL-1 and CCL-2 remained unchanged. (Figure 6A).

Strong correlations were found between the cytokine changes from day 0 to day 5 between CCL-5 and CCL-1, fractalkine and CCL-2, CCL-3, CCL-5, and CXCL-10, while strong reverse correlations were found between CXCL-10 and fractalkine, CCL-5 and CCL-3, and fractalkine and CCL-5 (Figure 6B).

**Figure 7:**
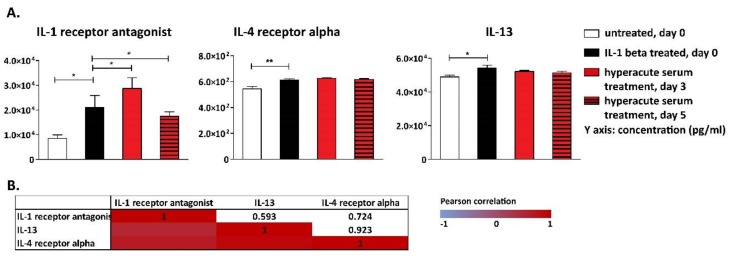
Anti-inflammatory cytokines detected in the tissue culture supernatant. IL-1β triggered an anti-inflammatory response evidenced by elevated interleukin-1 antagonist (IL-1RA), interleukin-4 receptor alpha (IL-4Rα), and IL-13 levels. IL-1RA significantly increased to day 3 in response to hyperacute serum treatment, but decreased until day 5, *n* = 12 (**A**). The significance level was *p* < 0.05, where * means that the *p*-value was between 0.05 and 0.01, ** means that *p*-value was between 0.01 and 0.001, and *** means that the *p*-value was lower than 0.001. The correlations of cytokine changes are presented in heatmap tables. Very strong correlations were found among all three anti-inflammatory cytokines (**B**).

The pattern of anti-inflammatory cytokines followed a similar course, i.e., IL-1β triggered an anti-inflammatory response evidenced by elevated IL-1RA, IL-4Rα and IL-13 levels. The key anti-inflammatory cytokine IL-1RA was significantly increased to day 3 in response to hyperacute serum treatment, which was subsequently reduced until day 5 (Figure 7A). Investigating the correlation between cytokine changes, very strong correlation was found between all the three anti-inflammatory cytokines (Figure 7B).

**Figure 8:**
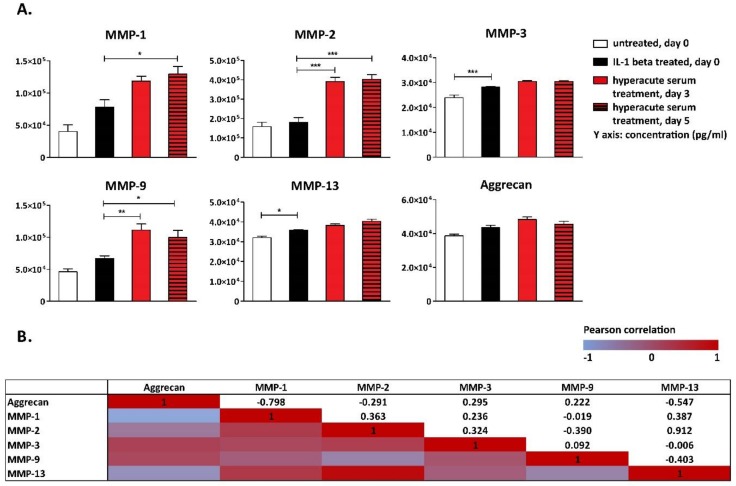

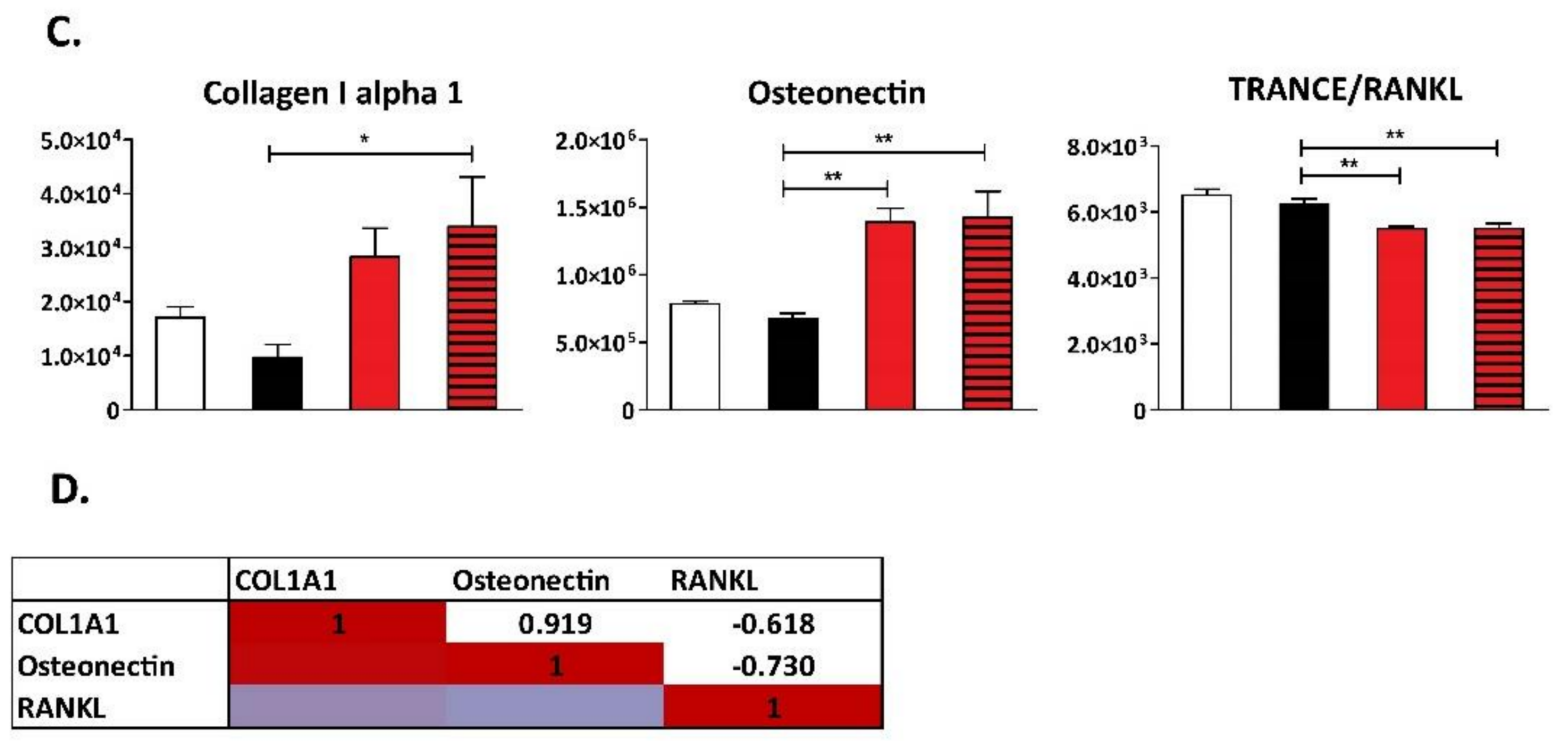
Biomarkers of tissue remodeling detected in tissue culture supernatant. IL-1β affected only MMP-3 and -13, while the concentrations of MMP-1, -2, and -9 increased significantly in response to hyperacute serum treatment (**A**). The significance level was *p* < 0.05, where * means that the *p*-value was between 0.05 and 0.01, ** means that the *p*-value was between 0.01 and 0.001, and *** means that the *p*-value was lower than 0.001, *n* = 12. The correlations of cytokine changes are presented in heatmap tables. A very strong correlation was found between MMP-2 and MMP-13, while reverse strong correlations were found between aggrecan and MMP-1 and MMP-13 (**B**). Osteonectin was also significantly elevated in response to hyperacute serum treatment. Similar changes were observed in collagen type I alpha I secretion, while receptor activator of nuclear factor kappa-B ligand (RANKL) continuously decreased in response to the hyperacute serum treatment (**C**). The significance level was *p* < 0.05, where * means that the *p*-value was between 0.05 and 0.01, ** means that *p*-value was between 0.01 and 0.001, and *** means that the *p*-value was lower than 0.001, *n* = 12. The correlation of cytokine changes between osteonectin and collagen 1 alpha 1 (COL1A1) was very strong, while reverse strong correlations were found between osteonectin, COL1A1, and RANKL, *n* = 12 (**D**).

Among MMPs, IL-1β increased MMP-3 and -13, while the concentrations of MMP 1, 2, and 9 all increased significantly in response to hyperacute treatment between day 0 and day 3; however, the concentration of MMPs were similar on day 3 and day 5 (Figure 8A). The concentration of aggrecan, which is the major proteoglycan that confers load-bearing properties to the cartilage, did not change significantly during the culture period (Figure 8A). While investigating the correlations among cytokine changes in response to hyperacute serum treatment, a very strong correlation was found between MMP-2 and MMP-13, while reverse strong correlations were found between aggrecan and MMP-1 and MMP-13 (Figure 8B).

Osteonectin, secreted mainly by regenerating chondrocytes, osteoblasts, and bone marrow progenitor cells, was significantly elevated in response to hyperacute serum treatment. Similar changes were observed in collagen type I alpha I (COL1A1) secretion, which is secreted mainly by osteoblasts and forms 90% of a bone’s extracellular matrix (Figure 8C). At the same time, the concentration of the osteoclastogenesis- and bone resorption-inducing RANKL continuously decreased in response to hyperacute serum treatment (Figure 8C). The correlation of cytokine changes between osteonectin and COL1A1 was very strong, while reverse strong correlations were found between osteonectin, COL1A1, and RANKL (Figure 8D).

Hyperacute serum contained much lower levels of the measured cytokines and MMPs than the supernatants, thus, changes in the cytokine level in the supernatant were not directly caused by the added cytokines presented in the hyperacute serum (Table S3).

## 4. Discussion

In the present study, we showed that it is possible to mimic the cytokine milieu of the osteoarthritic knee joint in an in vitro explant co-culture model. Stimulating the bone-cartilage-synovium tissues with IL-1β, we observed a sharp increase in the production of the downstream inflammatory mediators. However, when hyperacute serum was added, the balance tipped towards anti-inflammation.

Investigation of the pathomechanism of OA is a challenge for researchers in experimental and clinical orthopedics. Despite the fact that OA is considered to be a whole-joint disease, most published studies investigated a single cell type or, at most, two of the main tissues in vitro due to the technical limitations of classical culture methods [34,35,36]. However, cells in bone, cartilage, and synovial membranes communicate with each other through soluble mediators secreted into the synovial fluid, which can be detected and used as biomarkers for the progression of OA [37]. Culturing all three main tissues of the joint harvested from end-stage osteoarthritic human knees in co-culture allowed us to move one step closer to the clinical situations in an in vitro model. Remarkably, all 39 cytokines that were described in the synovial fluid as markers of OA have been promptly generated by this co-culture in a similar concentration as in vivo. Adding IL-1β as an inflammatory trigger further increased the downstream mediators, validating the method as reasonably similar to the in vivo situation. Concentration changes in response to IL-1β stimulation showed that the level of several, but far from all pro-inflammatory proteins increased, including IL-1β, TNF-α, IL-6Rα, and CXCL-8/IL-8, which are the most important inflammatory cytokines in OA. In response, anti-inflammatory proteins such as IL-1RA, IL-4Rα, and IL-13 also increased after IL-1β stimulation. In addition to interleukins, the extracellular matrix turnover enzymes MMP-3 and MMP-13 also increased and, together with the inhibition of collagen type I alpha I and osteonectin secretion, they contributed to the negative effect of IL-1β on cartilage and bone remodeling [38]. Taken together, our present data on a three-tissue osteoarthritic co-culture system are in agreement with the expected pathological cytokine composition of the joint, providing a suitable model for investigations on potential therapeutic models of OA.

Growth factors are considered to be the main components of platelet-rich blood derivatives, with the hypothesized action of cartilage regeneration [39]. However, a very thorough in vitro study has shown that PRP, which is rich in growth factors, does not support chondrogenic differentiation of mesenchymal stem cells [40]. Platelet-rich plasma is typically anti-coagulated by citrate or ethylenediamine-tetraacetic acid (EDTA) and then activated with thrombin and calcium, all of which have been shown to interfere with the healing process [26]. Hyperacute serum overcomes this concern, since it is basically the same as the physiological-activated plasma that is formed in a fresh wound. Our previous studies have pointed out that hyperacute serum is more proliferative than PRP despite the lower overall growth factor concentration [27,28,41,42]. It is reasonable to assume that the more physiological levels and ratios in hyperacute serum are better suited for the cells than over-concentrated and lysed PRP preparations. However, these measurements are typically performed on cell monolayers and it is advisable to extend the investigations to a model that closely resembles the in vivo situation before initiating clinical studies.

**Figure 9:**
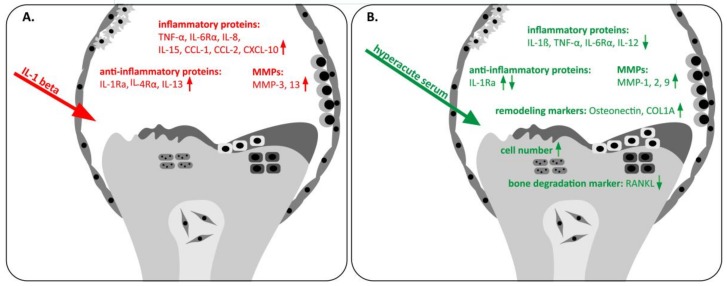
The effect of IL-1β stimulation and hyperacute serum treatment on osteoarthritic knee tissues. (**A**) IL-1β stimulation enhanced the level of inflammatory cytokines such as TNF-α, IL-6Rα, IL-8, IL-15, CCL-1, CCL-2, and CXCL-10 in an osteoarthritic tissue co-culture model, while the level of MMP-3 and MMP-13 also increased. (**B**) Hyperacute serum treatment reduced IL-1β, TNF-α, IL-6Rα, IL-12, and RANKL, while it increased the number of cells in all the three tissue types, MMP-1, -2, and -9, osteonectin, and COL1A1. The concentration of IL-1RA increased after adding hyperacute serum, but it also decreased at the end of the culture period.

Our current investigation about hyperacute serum effects on the cytokine milieu of an ex vivo osteoarthritic joint showed that serum treatment reduced inflammation and increased tissue viability (Figure 9). A large number of osteoarthritic inflammatory proteins and tissue remodeling markers were quantified to discover the way in which hyperacute serum affects the disease. Previous studies have typically used 2–5 selected cytokines and drew conclusions from the changes of these few molecules only, or mapped the overall cytokine content but did not present responses to treatment [43,44]. However, there is a lack of homogeneity in the published synovial cytokine data, neither the concentration ranges nor the changes to various treatments were comparable among different studies, making it impossible to draw a conclusion at this time [36,45]. Therefore, we created a custom two-plate multiplex protein array that contains 39 selected cytokines, chemokines, enzymes, extracellular matrix proteins, and even tissue degradation products to gain a more balanced view on the changes in the synovial milieu. In addition, the proliferation rate of the tissues was also measured, as this parameter cannot be reliably evaluated in vivo.

The cell proliferation of bone, cartilage, and synovial membrane was significantly elevated in response to hyperacute serum treatment compared to the human serum albumin treated-group, indicating that the cell proliferation rate was enhanced due to the growth factor content of hyperacute serum. The main inflammatory cytokines involved in the pathomechanism of OA, such as Il-1β, IL-6Rα, TNF-α, IL-12, and IL-17, decreased in response to hyperacute serum treatment, which resulted in a reduction of RANKL secretion leading to an inhibition of osteoclastogenesis and bone resorption [31,46]. A strong correlation was found between the decreasing trend of the main inflammatory cytokines in response to hyperacute serum treatment, meanwhile cell proliferation was strongly increasing in all the three tissue types. Most of the chemokines remained at similar levels with strongly increasing tissue viability during hyperacute serum treatment. Decreased concentrations of anti-inflammatory cytokines correlating with each other at the end of the experiment showed that inflammation was reduced to day 5 in response to hyperacute serum.

The primary enzymes responsible for cartilage and bone degradation in osteoarthritis are MMPs, most predominantly MMP-1, -2, -3, -9, and -13. Under normal conditions bone and cartilage remodeling is mediated by the balanced production of MMPs and other proteolytic enzymes, TIMPs, and by newly synthesized matrix proteins [47,48]. During the pathomechanism of OA, the increased level of inflammatory proteins, especially IL-1β, IL-6, and TNF-α, were found to elevate the level of MMPs and decrease the concentration of newly synthesized matrix proteins leading to enhanced tissue degradation [12,49,50].

Contradictory information is found in the literature about the concentration changes of MMPs in response to IL-1β treatment in vitro. On the basis of earlier studies, IL-1β induction increased the expression of MMP-1, -2, -3, -9, and -13 in cartilage cells [51,52,53], while Chubinskaya et al. [54] found significant increases in MMP-3 and -8, but not in MMP-1, -2, -13, and -14 expression after IL-1β stimulation. The stimulation of pannus-like cells and synovial plica cells by IL-1β increased the level of MMP-2 and -3, but not MMP-9 [55]. In mouse osteoblast cells, IL-1β caused a markedly enhanced expression of MMP-2, -3, and -14, while MMP-9 was absent [56].

On the basis of our present results, co-cultured human bone, cartilage, and synovium IL-1β stimulation increased the levels of MMP-3 and -13, while hyperacute serum increased MMP-1, -2, and -9 during the first 3 days, while, at the same time, decreased the level of inflammatory proteins. In addition, the level of MMP-1, -2, -3, -9, and -13 did not increase further between days 3 and 5 after hyperacute serum treatment, indicating that these changes were probably restricted to the first few days after application. At the same time, hyperacute serum enhanced the production of the tissue remodeling markers osteonectin and COL1A1, while it steadily decreased the secretion of RANKL during the culture period. A very strong correlation was found among the increases of osteonectin and COL1A1, and the reduction in RANKL [57]. Our present results obtained in an osteoarthritic joint explant model support the earlier observations in isolated cells that serum addition enhanced COL1A1 secretion from bone marrow explants and inhibited apoptosis [27]. Taken together, hyperacute serum can be a promising blood separation product in degenerative joint diseases, which enhances cell proliferation, the production of COL1A1 and osteonectin, while decreasing the level of RANKL, IL-1β, IL-6Rα, IL-12, and TNF-α (Figure 9). These changes in cell proliferation activity and the anti-inflammatory cytokine milieu indicate that hyperacute serum may have a beneficial effect on the OA knee joint.

## Supplementary Materials

The following are available online at https://www.mdpi.com/2073-4409/8/8/824/s1, Figure S1: Additional biomarkers of osteoarthritic inflammation. Table S1: The components of the co-culture supernatant and the osteoarthritic synovial fluid. Table S2: The protein concentrations in human serum albumin-treated co-culture supernatants. Table S3: The components of hyperacute serum and human serum albumin-containing medium.

## References

[1] Zhang Y., Jordan J.M. (2010). Epidemiology of Osteoarthritis. Clin. Geriatr. Med..

[2] Man G.S., Mologhianu G. (2014). Osteoarthritis pathogenesis—A complex process that involves the entire joint. J. Med. Life.

[3] Chen D., Shen J., Zhao W., Wang T., Han L., Hamilton J.L., Im H.-J. (2017). Osteoarthritis: Toward a comprehensive understanding of pathological mechanism. Bone Res..

[4] Daghestani H.N., Kraus V.B. (2015). Inflammatory Biomarkers in Osteoarthritis. Osteoarthr. Cartil..

[5] Hwang H.S., Kim H.A. (2015). Chondrocyte Apoptosis in the Pathogenesis of Osteoarthritis. Int. J. Mol. Sci..

[6] Miller R.E., Miller R.J., Malfait A.-M. (2014). Osteoarthritis Joint Pain: The Cytokine Connection. Cytokine.

[7] Scanzello C.R. (2017). Chemokines and Inflammation in Osteoarthritis: Insights from Patients and Animal Models. J. Orthop. Res..

[8] Beekhuizen M., Van Osch G., Bot A., Hoekstra M., Saris D., Dhert W., Creemers L. (2013). Inhibition of oncostatin M in osteoarthritic synovial fluid enhances GAG production in osteoarthritic cartilage repair. Eur. Cells Mater..

[9] Rose B.J., Kooyman D.L. (2016). A Tale of Two Joints: The Role of Matrix Metalloproteases in Cartilage Biology. Dis. Markers.

[10] Maruotti N., Corrado A., Cantatore F.P. (2017). Osteoblast role in osteoarthritis pathogenesis. J. Cell. Physiol..

[11] Tat S.K., Pelletier J.-P., Velasco C.R., Padrines M., Martel-Pelletier J. (2009). New perspective in osteoarthritis: The OPG and RANKL system as a potential therapeutic target?. Keio J. Med..

[12] Malemud C.J. (2015). The Biological Basis of Osteoarthritis: State of the Evidence. Curr. Opin. Rheumatol..

[13] Meszaros E., Malemud C.J. (2012). Prospects for treating osteoarthritis: Enzyme–protein interactions regulating matrix metalloproteinase activity. Ther. Adv. Chronic Dis..

[14] Lotz M., Martel-Pelletier J., Christiansen C., Brandi M.-L., Bruyere O., Chapurlat R., Collette J., Cooper C., Giacovelli G., Kanis J.A. (2013). Value of biomarkers in osteoarthritis: Current status and perspectives. Ann. Rheum. Dis..

[15] Sellam J., Berenbaum F. (2010). The role of synovitis in pathophysiology and clinical symptoms of osteoarthritis. Nat. Rev. Rheumatol..

[16] Wojdasiewicz P., Poniatowski, Łukasz A., Szukiewicz D. (2014). The Role of Inflammatory and Anti-Inflammatory Cytokines in the Pathogenesis of Osteoarthritis. Mediat. Inflamm..

[17] Bendinelli P., Matteucci E., Dogliotti G., Corsi M.M., Banfi G., Maroni P., Desiderio M.A. (2010). Molecular basis of anti-inflammatory action of platelet-rich plasma on human chondrocytes: Mechanisms of NF-κB inhibition via HGF. J. Cell. Physiol..

[18] O’Shaughnessey K., Matuska A., Hoeppner J., Farr J., Klaassen M., Kaeding C., Lattermann C., King W., Woodell-May J. (2014). An Autologous Protein Solution prepared from the blood of osteoarthritic patients contains an enhanced profile of anti-inflammatory cytokines and anabolic growth factors. J. Orthop. Res..

[19] Etulain J. (2018). Platelets in wound healing and regenerative medicine. Platelets.

[20] Filardo G., Kon E., Di Matteo B., Di Marino A., Sessa A., Merli M., Marcacci M. (2013). Leukocyte-poor PRP application for the treatment of knee osteoarthritis. Joints.

[21] Wasserman A., Matthewson G., Macdonald P. (2018). Platelet-Rich Plasma and the Knee—Applications in Orthopedic Surgery. Curr. Rev. Musculoskelet. Med..

[22] De Pascale M.R., Sommese L., Casamassimi A., Napoli C. (2015). Platelet Derivatives in Regenerative Medicine: An Update. Transfus. Med. Rev..

[23] Roffi A., Di Matteo B., Krishnakumar G.S., Kon E., Filardo G. (2017). Platelet-rich plasma for the treatment of bone defects: From pre-clinical rational to evidence in the clinical practice. A systematic review. Int. Orthop..

[24] Mariani E., Canella V., Cattini L., Kon E., Marcacci M., Di Matteo B., Pulsatelli L., Filardo G. (2016). Leukocyte-Rich Platelet-Rich Plasma Injections Do Not Up-Modulate Intra-Articular Pro-Inflammatory Cytokines in the Osteoarthritic Knee. PLoS ONE.

[25] Campbell K.A., Saltzman B.M., Mascarenhas R., Khair M.M., Verma N.N., Bach B.R., Cole B.J. (2015). Does Intra-articular Platelet-Rich Plasma Injection Provide Clinically Superior Outcomes Compared with Other Therapies in the Treatment of Knee Osteoarthritis? A Systematic Review of Overlapping Meta-analyses. Arthrosc. J. Arthrosc. Relat. Surg..

[26] Kardos D., Simon M., Vácz G., Hinsenkamp A., Holczer T., Cseh D., Sárközi A., Szenthe K., Bánáti F., Szathmary S. (2019). The Composition of Hyperacute Serum and Platelet-Rich Plasma Is Markedly Different despite the Similar Production Method. Int. J. Mol. Sci..

[27] Simon M., Major B., Vácz G., Kuten O., Hornyak I., Hinsenkamp A., Kardos D., Bagó M., Cseh D., Sarkozi A. (2018). The Effects of Hyperacute Serum on the Elements of the Human Subchondral Bone Marrow Niche. Stem Cells Int..

[28] Kuten O., Simon M., Hornyák I., De Luna-Preitschopf A., Nehrer S., Lacza Z. (2018). The Effects of Hyperacute Serum on Adipogenesis and Cell Proliferation of Mesenchymal Stromal Cells. Tissue Eng. Part A.

[29] Kardos D., Hornyák I., Simon M., Hinsenkamp A., Marschall B., Várdai R., Kállay-Menyhárd A., Pinke B., Mészáros L., Kuten O. (2018). Biological and Mechanical Properties of Platelet-Rich Fibrin Membranes after Thermal Manipulation and Preparation in a Single-Syringe Closed System. Int. J. Mol. Sci..

[30] Mabey T., Honsawek S. (2015). Cytokines as biochemical markers for knee osteoarthritis. World J. Orthop..

[31] Jay P.R., Centrella M., Lorenzo J., Bruce A.G., Horowitz M.C. (1996). Oncostatin-M: A new bone active cytokine that activates osteoblasts and inhibits bone resorption. Endocrinology.

[32] Maldonado M., Nam J. (2013). The Role of Changes in Extracellular Matrix of Cartilage in the Presence of Inflammation on the Pathology of Osteoarthritis. BioMed Res. Int..

[33] Yuan Q., Sun L., Li J.-J., An C.-H. (2014). Elevated VEGF levels contribute to the pathogenesis of osteoarthritis. BMC Musculoskelet. Disord..

[34] Johnson C.I., Argyle D.J., Clements D.N. (2016). In vitro models for the study of osteoarthritis. Vet. J..

[35] Otero M. (2018). In vitro OA models to study chondrocytes and cartilage. Osteoarthr. Cartil..

[36] Tsuchida A.I., Beekhuizen M., Hart M.C.T., Radstake T.R., Dhert W.J., Saris D.B., Van Osch G.J., Creemers L.B. (2014). Cytokine profiles in the joint depend on pathology, but are different between synovial fluid, cartilage tissue and cultured chondrocytes. Arthritis Res. Ther..

[37] Ding J., Niu X., Su Y., Li X. (2017). Expression of synovial fluid biomarkers in patients with knee osteoarthritis and meniscus injury. Exp. Ther. Med..

[38] Goldring M.B., Otero M. (2011). Inflammation in osteoarthritis. Curr. Opin. Rheumatol..

[39] Kennedy M.I., Whitney K., Evans T., Laprade R.F. (2018). Platelet-Rich Plasma and Cartilage Repair. Curr. Rev. Musculoskelet. Med..

[40] Liou J.-J., Rothrauff B.B., Alexander P.G., Tuan R.S. (2018). Effect of Platelet-Rich Plasma on Chondrogenic Differentiation of Adipose-and Bone Marrow-Derived Mesenchymal Stem Cells. Tissue Eng. Part A.

[41] Jeyakumar V., Niculescu-Morzsa E., Bauer C., Lacza Z., Nehrer S. (2017). Platelet-Rich Plasma Supports Proliferation and Redifferentiation of Chondrocytes during In Vitro Expansion. Front. Bioeng. Biotechnol..

[42] Vácz G., Major B., Gaál D., Petrik L., Horváthy D.B., Han W., Holczer T., Simon M., Muir J.M., Hornyák I. (2018). Hyperacute serum has markedly better regenerative efficacy than platelet-rich plasma in a human bone oxygen–glucose deprivation model. Regen. Med..

[43] Vangsness C.T., Burke W.S., Narvy S.J., Macphee R.D., Fedenko A.N. (2011). Human knee synovial fluid cytokines correlated with grade of knee osteoarthritis—A pilot study. Bull. NYU Hosp. Jt. Dis..

[44] Anitua E., Sánchez M., De La Fuente M., Azofra J., Zalduendo M., Aguirre J.J., Andía I. (2009). Relationship between Investigative Biomarkers and Radiographic Grading in Patients with Knee Osteoarthritis. Int. J. Rheumatol..

[45] Jayadev C., Snelling S., Price A., Hulley P. (2013). Multiplex analysis of osteoarthritic synovial fluid: A comparison of Luminex & Mesoscale discovery. Osteoarthr. Cartil..

[46] Amarasekara D.S., Yun H., Kim S., Lee N., Kim H., Rho J. (2018). Regulation of Osteoclast Differentiation by Cytokine Networks. Immune Netw..

[47] Page-McCaw A., Ewald A.J., Werb Z. (2007). Matrix metalloproteinases and the regulation of tissue remodelling. Nat. Rev. Mol. Cell Boil..

[48] Streuli C. (1999). Extracellular matrix remodelling and cellular differentiation. Curr. Opin. Cell Boil..

[49] Burrage P.S., Mix K.S., Brinckerhoff C.E. (2006). Matrix metalloproteinases: Role in arthritis. Front. Biosci. J. Virtual Libr..

[50] Vuolteenaho K., Koskinen-Kolasa A., Laavola M., Nieminen R., Moilanen T., Moilanen E. (2017). High synovial fluid interleukin-6 levels are associated with increased matrix metalloproteinase levels and severe radiographic changes in osteoarthritis patients. Osteoarthr. Cartil..

[51] Mengshol J.A., Vincenti M.P., Coon C.I., Barchowsky A., Brinckerhoff C.E. (2000). Interleukin-1 induction of collagenase 3 (matrix metalloproteinase 13) gene expression in chondrocytes requires p38, c-Jun N-terminal kinase, and nuclear factor kappaB: Differential regulation of collagenase 1 and collagenase 3. Arthritis Rheum..

[52] Xie J., Fu N., Cai L.-Y., Gong T., Li G., Peng Q., Cai X.-X. (2015). The effects of interleukin-1β in modulating osteoclast-conditioned medium’s influence on gelatinases in chondrocytes through mitogen-activated protein kinases. Int. J. Oral Sci..

[53] Richardson D.W., Dodge G.R. (2000). Effects of interleukin-1beta and tumor necrosis factor-alpha on expression of matrix-related genes by cultured equine articular chondrocytes. Am. J. Vet. Res..

[54] Chubinskaya S., Kuettner K.E., Cole A.A. (1999). Expression of matrix metalloproteinases in normal and damaged articular cartilage from human knee and ankle joints. Lab. Investig..

[55] Yang C.-C., Lin C.-Y., Wang H.-S., Lyu S.-R. (2013). Matrix Metalloproteases and Tissue Inhibitors of Metalloproteinases in Medial Plica and Pannus-like Tissue Contribute to Knee Osteoarthritis Progression. PLoS ONE.

[56] Kusano K., Miyaura C., Inada M., Tamura T., Ito A., Nagase H., Kamoi K., Suda T. (1998). Regulation of Matrix Metalloproteinases (MMP-2, -3, -9, and -13) by Interleukin-1 and Interleukin-6 in Mouse Calvaria: Association of MMP Induction with Bone Resorption. Endocrinology.

[57] Cao X. (2018). RANKL-RANK signaling regulates osteoblast differentiation and bone formation. Bone Res..

