# Supplementary material for: Investigation of Cytokine Changes in Osteoarthritic Knee Joint Tissues in Response to Hyperacute Serum Treatment
Source: Cells. 2019 Aug 3;8(8):824. doi: 10.3390/cells8080824 (PMC6721638; doi:10.3390/cells8080824)
Supplement: Supplementary file 1 [file cells-08-00824-s001.pdf]

cells MDPI

A.

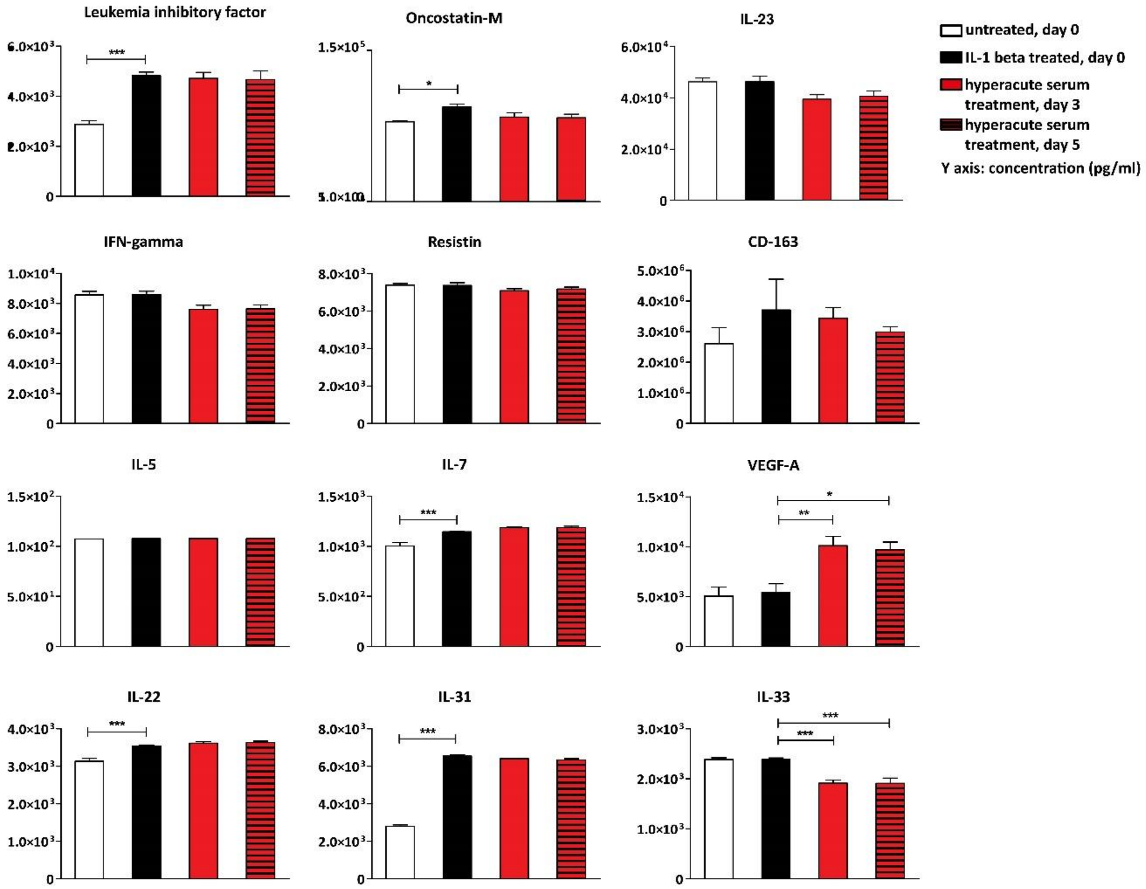

B.

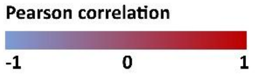

	CD-163	IFN-gamma	IL-23	IL-31	IL-33	LIF	Oncostatin-M	Resistin	IL-5	IL-7	IL-22	VEGF
CD-163	1	0.532	-0.179	-0.085	0.579	-0.400	-0.268	-0.058	-0.180	-0.220	-0.686	0.351
IFN-gamma		1	0.448	0.432	0.962	0.281	0.481	0.789	0.148	-0.379	-0.156	-0.184
IL-23			1	0.849	0.545	0.478	0.624	0.601	0.590	0.050	0.457	0.159
IL-31				1	0.462	0.749	0.841	0.647	0.810	0.093	0.273	-0.071
IL-33					1	0.135	0.370	0.688	0.223	-0.214	-0.066	0.078
LIF						1	0.967	0.736	0.586	-0.137	0.224	-0.695
Oncostatin-M							1	0.858	0.655	-0.113	0.266	-0.592
Resistin								1	0.435	-0.222	0.268	-0.557
IL-5									1	0.625	0.552	0.018
IL-7										1	0.668	0.399
IL-22											1	0.021
VEGF												1

Figure S1. Additional biomarkers of osteoarthritic inflammation. The level of leukemia inhibitory factor, oncostatin-M, Il-7, IL-13, IL-22 and IL-31 was significantly elevated in response to IL-1β treatment. Hyperacute serum affected only the concentration of VEGF-A and IL-33 (Part A.), n= 12. The significance level was $p < 0.05$, where * means that p is between 0.01 and 0.05, ** means that p is between 0.01 and 0.001, and *** means that p is lower than 0.001. Investigating the correlation of changes between day 0 and day 5, strong correlation was found between numerous pairs of cytokines. We considered the correlation very strong if $r>0.75$, and strong if $0.75>r>0.5$ (Part B.), n=12.

Supplementary Table 1. The components of the co-culture supernatant and the osteoarthritic synovial fluid. Mean concentration of each cytokine in the two kind of samples was presented.

Cytokines	*Mean of concentration (ng/ml)*	
	**in vitro (ng/ml)**	**in vivo (ng/ml)**
CD-163	3706,666	2990,840
SPARC/Osteonectin	676,268	1421,979
MMP-2	181,201	402,497
Fractalkine	116,307	100,425
OSM	94,744	83,848
MMP-1	78,103	129,484
CXCL-8/IL-8	77,535	81,125
MMP-9	67,068	99,811
CCL-3/MIP1a	58,194	54,371
CCL-2/MCP-1	56,880	60,093
IL-13	54,276	51,338
IL-23	46,271	40,690
Aggrecan	43,427	45,421
MMP-13	35,785	40,323
MMP-3	28,135	30,413
IL-1RA	21,041	17,403
IL1-β	17,401	2,772
IL-2	12,035	11,226
IL-6Rα	10,535	40,776
COL1A1	9,697	33,893
IL-12	9,550	9,157
IFN-γ	8,576	7,638
IL-18	7,648	7,549
Resistin	7,373	7,181
IL-31	6,541	6,338
TRANCE/RANKL	6,246	5,494
VEGF-A	5,430	9,690
LIF	4,821	4,669
IL-22	3,545	3,545
CCL-5/Rantes	3,361	5,966
EpCAM/TROP1	2,916	3,296

IL-33	2,386	1,909
IL-17A	1,624	1,356
IL-7	1,146	1,189
CXCL-10/IP-10	0,970	0,754
TNF-α	0,857	0,607
IL-4Rα	0,612	0,618
IL-10	0,511	0,427
CCL-1/I-309	0,434	0,416
IL-15	0,331	0,329
IL-5	0,108	0,108

Supplementary Table 2. The protein concentrations in human serum albumin treated co-culture supernatants. The mean concentration and standard error of mean (SEM) of each protein, measured on day 3 and day 5 was presented in this table.

Cytokines	*Mean of concentration (pg/ml)*			
	**Albumin, control day 3 (pg/ml)**	**SEM**	**Albumin, control day 5 (pg/ml)**	**SEM**
Aggrecan	41 791	2 601	44 170	1 659
CCL-1/I-309	417	9	403	5
CCL-2/MCP-1	36 341	6 961	28 249	6 558
CCL-3/MIP1a	56 344	1 022	55 415	1 014
CD-163	2 359 511	302 351	2 080 235	145 780
COL1A1	9 384	2 226	15 946	10 764
Fractalkine	101 186	2 412	96 228	1 642
CXCL-10/IP-10	584	33	563	54
IFN-γ	8 494	239	8 281	190
IL1-β	5 368	1 485	2 155	561
IL-1RA	16 091	3 007	15 433	3 399
IL-2	11 310	199	11 443	270
IL-6Rα	8 596	945	13 364	5 600
IL-10	421	7	417	5
IL-12 p70	9 148	134	9 286	140
IL-13	49 752	687	47 954	734
IL-15	300	6	296	12
IL-17A	1 409	193	1 276	165
IL-18	7 405	88	7 578	100
IL-23	45 007	1 667	43 442	1 273

IL-31	6 253	48	6 140	73
IL-33	2 375	73	2 246	57
LIF	3 729	108	3 266	204
MMP-1	76 628	6 559	84 505	16 452
MMP-2	202 509	13 385	228 459	54 832
OSM	82 078	2 117	77 915	1 974
Resistin	7 222	132	7 146	107
SPARC/Osteonectin	594 120	46 465	675 913	240 283
TNF-α	590	27	596	32
TRANCE/RANKL	8 604	2 492	6 188	119
CCL-5/Rantes	3 277	263	3 517	339
CXCL-8/IL-8	75 482	2 613	73 968	2 455
IL-4Rα	614	2	592	7
IL-5	108	0	108	0
IL-7	1 155	6	1 136	14
IL-22	3 531	10	3 447	38
MMP-3	28 697	247	27 820	568
MMP-9	79 403	11 364	78 537	15 459
MMP-13	36 895	731	36 608	622
VEGF-A	5 457	567	5 740	755

Supplemenatry Table 3. The components of hyperacute serum and human serum albumin containing medium. The mean concentration of the measured proteins in 10% hyperacute serum, and 5% human serum albumin containing culture medium was presented in this table.

Cytokines	*Mean of concentration (pg/ml)*	
	**Hyperacute serum containing medium (pg/ml)**	**Human serum albumin contaning medium (pg/ml)**
Aggrecan	6785,508	198,682
CCL-1/I-309	19,270	0,000
CCL-2/MCP-1	200,819	21,508
CCL-3/MIP1a	2881,027	5,559
CD-163	135213,974	1091,271
COL1A1	360,290	32,691
Fractalkine	4649,019	120,716
CXCL-10/IP-10	17,349	0,485
IFN-γ	438,833	0,583
IL1-β	44,376	1,526

IL-1RA	200,674	1,225
IL-2	614,999	8,006
IL-6Rα	3097,122	268,567
IL-10	19,517	1,774
IL-12	459,922	33,039
IL-13	2410,747	218,770
IL-15	12,111	1,075
IL-17A	87,949	0,000
IL-18	382,946	8,196
IL-23	2412,957	2,225
IL-31	304,853	1,133
IL-33	104,366	0,881
LIF	113,073	0,000
MMP-1	204,341	16,157
MMP-2	9416,864	1360,354
OSM	3268,440	266,980
Resistin	361,025	50,539
SPARC/Osteonectin	40571,472	180,257
TNF-α	24,744	2,064
TRANCE/RANKL	311,739	3,095
CCL-5/Rantes	588,443	92,465
CXCL-8/IL-8	7,277	2,464
IL-4Rα	0,938	4,788
IL-5	5,339	0,000
IL-7	8,852	1,378
IL-22	105,286	8,693
MMP-3	76,393	4,783
MMP-9	3967,262	251,319
MMP-13	45,656	0,000
VEGF-A	101,643	23,477
